# Expanding the clinical spectrum of DNASE1L3-associated monogenic lupus: A case series of 4 syrian pediatric cases

**DOI:** 10.1097/MD.0000000000050283

**Published:** 2026-08-14

**Authors:** Alyamama Kousa, Ahmad Alhamwi, Mohammad Khaled Alsayed, Sara Jabaly, Basheer Khalil

**Affiliations:** a Department of Cardiology, Syrian Arab Red Crescent Hospital, Damascus, Syrian Arab Republic; b Department of Cardiology, Damascus Hospital, Damascus, Syrian Arab Republic; c Department of Otorhinolaryngology, Damascus Hospital, Damascus, Syrian Arab Republic; d Damascus University, Faculty of Medicine, Damascus, Syrian Arab Republic; e Rheumatology Pediatric Department, Children’s Damascus University Hospital, Damascus, Syrian Arab Republic.

**Keywords:** *DNASE1L3* deficiency, hypocomplementemic urticarial vasculitis, IgA glomerulonephritis, monogenic lupus, pediatric autoimmune disease

## Abstract

**Rationale:**

*DNASE1L3* deficiency is a rare autosomal-recessive monogenic form of systemic lupus erythematosus, characterized by defective clearance of extracellular DNA, leading to immune-complex formation, autoantibody production, and systemic inflammation. While early-onset lupus nephritis and hypocomplementemic urticarial vasculitis are hallmark features, the full clinical spectrum remains incompletely understood, particularly in pediatric populations.

**Patient concerns and diagnoses:**

We report 4 cases from 2 unrelated consanguineous Syrian families, including 1 genetically confirmed case with a pathogenic DNASEI L3 variant, 1 case harboring a homozygous DNASEI L3 variant of uncertain significance, and 2 phenotypically concordant siblings without genetic testing. Clinical, laboratory, and histopathologic data were reviewed to characterize disease manifestations, organ involvement, serologic profiles, and therapeutic outcomes.

**Interventions and outcomes:**

All patients presented with recurrent fever, cutaneous rash, and musculoskeletal involvement, with notable variability in autoantibody profiles, renal pathology, and disease severity. Genetic analysis identified a homozygous pathogenic DNASEI L3 variant in case 3 and a homozygous DNASEI L3 variant of uncertain significance in case 1, supporting a clinically suspected DNASEI L3-associated monogenic lupus-spectrum disease. Renal involvement ranged from IgA glomerulonephritis to class IV lupus nephritis. Ocular manifestations, including conjunctival congestion and papilledema, were observed in 3 patients, highlighting an underrecognized feature. Therapeutic responses varied: 1 patient remained stable on baricitinib, while 2 patients succumbed to severe neurologic or renal complications. This series underscores the heterogeneous phenotypic spectrum of DNASEI L3-associated disease, including atypical seronegative presentations and diverse renal pathology.

**Lessons:**

DNASEI L3-associated disease should be considered in children with early-onset vasculitic rash, hypocomplementemia, and nephritis, regardless of classical autoantibody status. Our cases expand the known clinical spectrum, emphasize the potential for ocular involvement, and suggest a role for targeted therapies such as JAK inhibitors in interferon-driven disease. Early genetic testing is essential for timely diagnosis and management of this potentially life-threatening condition.

**Key Points**

1. This case series broadens the known clinical spectrum of pediatric *DNASE1L3* deficiency.
2. Genetic testing is crucial in early-onset lupus-like disease, even when classical autoantibodies are absent.
3. Renal involvement is heterogeneous, ranging from IgA nephropathy to class IV lupus nephritis.
4. Ocular manifestations, including conjunctival congestion and papilledema, are underrecognized in *DNASE1L3* deficiency.
5. Baricitinib demonstrated potential therapeutic benefit, highlighting the role of *JAK* inhibition in this interferon-driven disease.

## 1. Introduction

Deoxyribonuclease 1-like 3 (*DNASE1L3*) is a secreted endonuclease essential for the degradation of extracellular DNA released from apoptotic cells and neutrophil extracellular traps.^[1,2]^ It belongs to the *DNASE1* family but has superior activity in digesting chromatin, thereby preventing the accumulation of circulating nucleic acids that can trigger autoimmunity.^[1,3]^ *DNASE1L3* is primarily expressed by dendritic cells and macrophages in the liver, spleen, and gut, maintaining immune homeostasis through clearance of cell-free DNA.^[3]^ Loss-of-function mutations in *DNASE1L3* cause defective clearance of extracellular chromatin, leading to immune-complex formation, production of anti-DNA antibodies, and a distinct monogenic form of systemic lupus erythematosus (SLE).^[4]^ Neutralizing autoantibodies against *DNASE1L3* have also been identified in sporadic lupus, contributing to reduced enzymatic activity.^[5]^ Clinically, *DNASE1L3* deficiency presents as an autosomal-recessive, childhood-onset autoimmune condition characterized by lupus nephritis, hypocomplementemic urticarial vasculitis, and positive ANCA, with occasional pulmonary or gastrointestinal involvement.^[1,6]^ The phenotype is heterogeneous, affecting renal, hematologic, cutaneous, and musculoskeletal systems. Common variants within the *DNASE1L3* locus are additionally associated with increased susceptibility to polygenic autoimmune diseases such as SLE, systemic sclerosis, and rheumatoid arthritis.^[7]^ Since the initial description by Al-Mayouf et al,^[4]^ multiple families and cohorts including recent Omani and Emirati studies have expanded the known clinical spectrum of *DNASE1L3* deficiency.^[8,9]^ However, the disorder remains underrecognized, particularly in pediatric patients with lupus-like manifestations and negative or atypical serologic profiles. In this report, we describe 4 children from 2 unrelated consanguineous Syrian families with genetically confirmed or clinically suspected DNASEI L3-associated disease. One patient harbored a pathogenic homozygous DNASEI L3 variant, while another carried a homozygous variant of uncertain significance (VUS) in the setting of a highly suggestive clinical phenotype. The remaining 2 cases were phenotypically concordant siblings without available genetic testing. Each patient exhibited variable combinations of fever, rash, arthralgia, and renal involvement. Through these cases, we aim to enrich current understanding of this rare monogenic lupus, emphasize its phenotypic diversity, and highlight the importance of early genetic testing for children with atypical or seronegative lupus presentations.

## 2. Case presentation

In this article, we describe 4 pediatric cases from 2 unrelated consanguineous Syrian families, including 2 sibling pairs: cases 1 and 2 (first family) and cases 3 and 4 (second family). Case 3 carried a genetically confirmed pathogenic DNASEI L3 variant, whereas case 1 harbored a homozygous DNASEI L3 VUS in the setting of a highly suggestive clinical phenotype. Cases 2 and 4 did not undergo genetic testing but demonstrated marked phenotypic concordance with their affected siblings.

### 2.1. Case 1

A 5-year-old Syrian boy was admitted to the rheumatology department complaining of a non-pruritic rash, pain and swelling of the hands, legs, and fingers, redness of the eyes, and recurrent fever for 4 years. His parents were first-degree cousins, and his aunt had allergic eczema. Physical examination revealed moderate pallor, a diffuse non-pruritic maculopapular erythematous rash across the entire body (appearing suddenly and resolving within 12 hours), cervical and axillary lymphadenopathy (3–4 cm), and hepatosplenomegaly. Laboratory evaluation revealed microcytic anemia, thrombocytosis, elevated erythrocyte sedimentation rate (ESR), and decreased serum complement components C3 and C4. Peripheral blood smear showed microcytic hypochromic anemia and rouleaux formation. Urinalysis demonstrated hemoglobinuria, erythrocytes > 200 cells/mL, microalbuminuria, and anti-dsDNA titer of 1:40. Immunological tests, including anticardiolipin antibody and anti-Smith antibody, were negative, whereas anti-dsDNA and antinuclear antibody (ANA) were positive. IgA, IgG, and IgE levels were elevated, and the direct Coombs test was weakly positive. Flow cytometry revealed elevated CD3 (2971.8) and CD3+ CD8 (1774.6) levels. Abdominal ultrasound confirmed splenomegaly, and ophthalmologic examination detected conjunctival congestion and severe hyperopia. Cervical lymph node biopsy showed diffuse follicular hyperplasia, and renal biopsy was consistent with IgA glomerulonephritis. He was treated with corticosteroids without improvement, followed by ciclosporin and angiotensin-converting enzyme inhibitors. Whole-exome sequencing identified a homozygous DNASEI L3 VUS. Although the molecular findings were insufficient to establish a definitive genetic diagnosis according to ACMG criteria, the combination of consanguinity, early-onset disease, hypocomplementemia, nephritis, systemic inflammatory manifestations, and familial clustering strongly suggested a DNASEI L3-associated monogenic lupus-spectrum disorder.During follow-up, he suffered from a sudden seizure and died.

### 2.2. Case 2

A 2-year-and-7-month-old Syrian boy was admitted to the rheumatology department complaining of a non-pruritic rash, pain and swelling of the hands, legs, and fingers, redness of the eyes, and recurrent fever for 11 months. Physical examination revealed moderate pallor, a diffuse non-pruritic maculopapular erythematous rash (sparing the face), which appeared suddenly starting in the lower extremities, progressing to the trunk, and then to the upper extremities, resolving within 12 hours; restricted joint movement; submandibular, axillary, and inguinal lymphadenopathy; and hepatomegaly. Laboratory evaluation showed microcytic anemia, elevated ESR, decreased serum complement component C3, and a weakly positive direct Coombs test. Peripheral blood smear confirmed microcytic hypochromic anemia and rouleaux formation. Immunological tests, including anti-dsDNA antibody, ANA, and anti-Smith antibody, were negative. Ophthalmologic examination revealed conjunctival congestion and grade 1 papilledema. He was treated with corticosteroids and became corticosteroid-dependent, followed by ciclosporin. During follow-up, he remained clinically stable. Although genetic testing was not available for this patient, the marked phenotypic similarity to case 1 and the familial relationship raised strong suspicion for a shared inherited autoimmune disorder.

### 2.3. Case 3

A 5-year-old Syrian girl presented to the rheumatology department with a 1.5-year history of recurrent non-pruritic urticaria and an erythematous maculopapular rash affecting her hands, legs, and abdomen. She also reported frequent eye redness (three episodes in the past 3 weeks). The patient’s parents are consanguineous (first-degree cousins). Over the past 2 weeks, the eye redness was accompanied by swelling of her left heel and ankle. Physical examination revealed profuse pigmented skin lesions, chronic idiopathic urticaria, a rash localized to both lower extremities (notably the left thigh and right foot), and 1 × 2 cm axillary lymphadenopathy. Ocular examination showed conjunctival injection, predominantly in the right eye. Laboratory findings revealed anemia, crystalluria, decreased serum ferritin, mean corpuscular hemoglobin concentration, mean corpuscular volume, and mean platelet volume; decreased serum complement C3 and C4; elevated ESR; eosinophilic and neutrophilic dermal infiltrates; lymphocytosis and neutropenia; superficial dermal perivascular inflammatory infiltrate; thrombocytosis; and evidence of vascular skin abnormality and vasculitis The patient’s brother died during childhood following a diagnosis of stage IV chronic kidney disease and SLE. Genetic analysis identified a homozygous pathogenic DNASEI L3 variant consistent with autosomal-recessive SLE type 16. Current medication: prednisolone 3 mL daily and Folic acid and iron supplementation.

### 2.4. Case 4

A 3.5-year-old Syrian boy was admitted to the rheumatology department with a 1.5-year history of pruritic rash, pain in the trunk and extremities, recurrent fever, and hypertension managed with medication. Physical examination revealed severe pallor, eyelid edema, a diffuse pruritic maculopapular erythematous rash on the trunk and extremities (blanching on pressure), submandibular, retroauricular, axillary, and inguinal lymphadenopathy, and splenomegaly. Laboratory evaluation revealed microcytic anemia, thrombocytosis, elevated ESR and uremia. His urine analysis showed hemoglobinuria, erythrocytes (130–150) cell per mL, leukocytes^[8-16]^ cell per mL and proteinuria. 24-hour urine collection: Glomerular filtration rate of 59 mL/min/1.73 m^2^, 622 mg/dL protein, and 63 mg/dL creatinine. His immunological tests including cardiolipin antibody IgM, anti-smith, anti-DNA (ds), ANA, cytoplasmic anti-neutrophil cytoplasmic antibody (c-ANCA) and perinuclear anti-neutrophil cytoplasmic antibody (p-ANCA) were positive. While IgA, IgG and IgM were elevated, and indirect Coombs test was positive. Abdominal ultrasound showed hepatomegaly and bilateral renal hyperechogenicity whereas echocardiogram revealed concentric hypertrophy of the ventricular septum. Renal biopsy demonstrated diffuse proliferative glomerulonephritis, consistent with Class IV lupus nephritis. He was treated with Corticosteroid, Cyclophosphamide and Chloroquine, Aspirin and Metoprolol. At the follow-up, he suffered from stage 4 chronic kidney disease and died. Although molecular confirmation was unavailable, the clinical overlap with case 3 and the familial background strongly supported a shared monogenic lupus-spectrum disorder.

Laboratory evaluations of the 4 cases are summarized in Table 1.

**Table 1:** Laboratory evaluation results for the 4 cases studied.

Investigation	Result				Units	Reference range
	Case 1	Case 2	Case3	Case 4		
Red blood cells	4.62	3.53	4.48	3.53	×10.e3/µL	3.5–5.5
Hemoglobin	9.5	6	9.3	7.4	g/dL	15.5–16.5
HCT	29.3	19.4	32.7	23	%	35–55
MCV	63.3	54.9	74	57	fL	75–100
MCH	20.7	17	23.4	19.8	pg	25–35
reticulocytes	–	1%	–	–	%	0.5–1.5
White blood cells	8.3	10.5	8100	10,300	×10.e3/µL	3.5–10
Lymphocytes	46.9	56.1	64	31	%	15–50
Granulocytes	46	30.7	35	62	%	35–80
Platelet	505	191	298	602	×10.e3/µL	100–400
Sodium	136	–	–	139	mEq/L	135–145
Potassium	4.46	–	–	4.2	mEq/L	3.4–4.7
Phosphate	–	5.48	–	–	mg/dL	2.7–6.2
ALT/GPT	9	6	20	11.7	U/L	10–50
AST/GOT	24	30	–	–	U/L	8.33
ALP	125	–	–	–	U/L	<187
Total protein	6.3	7.8	7	4.74	g/dL	6–8.3
Albumin	3.3	4.3	3.71	2.44	g/dL	5.4–3.6
LDH	333	412	–	345	mg/dL	105–333
CRP	4.2	2.9	2.3	24	mg/dL	0.3–1
ESR	70	65	21	66	mm/1st h	0–20
TG	122	–	–	–	mg/dL	150>
cholesterol	105	–	–	–	mg/dL	200>
Urea	25	22	27	66	mg/dL	5–20
Creatinine	0.4	0.5	0.32	0.7	mg/dL	0.3–0.7
Uric acid	4.6	4.44	–	–	mg/dL	2.5–5.5
C3 complement	72.48	56.76	75	120	mg/dL	75–175
Complement C4	7.85	9.71	12	30	mg/dL	14–36
VonWillebrand factor (antigen)	119	–	–	342	%	50–160
Anti cardiolipin IgG Antibodies	8.4	–	–	Negative	U/mL	10<
Anti cardiolipin IgM Antibodies	3.6	–	–	Positive	U/mL	10<
Rheumatoid factor	8.9	–	–	10	IU/mL	0–24
Anti-DNA (ds)	Positive at dilution 1/20	Negative	negative	positive	–	Negative
Anti Smith	6.8	1.9	Negative	99.1	U/mL	Negative: up to 18 Positive more than 18
ANA	Positive (Homogenous) at dilution 1/40	Negative	–	Positive	–	Negative
Immunoglobulin A IgA	3.27	–	–	134	IU/mL	0.27–1.95
Immunoglobulin M IgM	1.57	–	–	314	IU/mL	0.5–2
Immunoglobulin G IgG	16.76	–	–	500.4	IU/mL	5–14
Immunoglobulin E IgE	87.8	–	–	–	IU/mL	60<
HCV antibody	0.25	–	0-198	–	Log IU/mL	Negative: up to 1 Positive more than 1
HBs Ag	Negative	–	–	–	–	Negative

µL = microliter, ALP = alkaline phosphatase, ALT/GPT = alanine aminotransferase/glutamic-pyruvic transaminase, ANA = antinuclear antibody, AST/GOT = aspartate aminotransferase/glutamic-oxalacetat transaminase, CRP = C-reactive protein, ESR = erythrocyte sedimentation rate, fL = femtoliter, g/dL = gram/deciliter, HBs Ag = Hepatitis B Surface Antigen, HCT = hematocrit, HCV = hepatitis C virus, IgA = immunoglobulin A, IgE = immunoglobulin E, IgG = immunoglobulin G, IgM = immunoglobulin M, JAK = Janus Kinase, LDH = lactate dehydrogenase, MCH = mean corpuscular hemoglobin, MCV = mean corpuscular volume, mEq/L = milliequivalents per liter, pg = picograms, TG = triglyceride, U/L = units per liter.

## 3. Discussion

We present 4 pediatric cases from 2 unrelated consanguineous Syrian families, including one genetically confirmed case with a pathogenic DNASEI L3 variant, one patient harboring a homozygous DNASEI L3, and 2 phenotypically concordant siblings without molecular testing. These cases expand the clinical spectrum of *DNASEI L3*-associated monogenic lupus-spectrum disease and highlight several unique features that distinguish them within the global literature.

*DNASE1L3* encodes a secreted nuclease essential for the clearance of extracellular chromatin, particularly microparticle-bound DNA and neutrophil extracellular traps. Loss-of-function mutations impair DNA degradation, promoting antinuclear autoimmunity and systemic inflammation.^[1]^ The initial description by Al-Mayouf et al.^[4]^ established autosomal-recessive *DNASE1L3* deficiency as a cause of early-onset SLE, with a high prevalence of lupus nephritis. Subsequent work demonstrated that the disorder also overlaps with hypocomplementemic urticarial vasculitis syndrome (HUVS), reinforcing a disease spectrum rather than discrete entities.^[10]^

In the largest pediatric cohort to date, Abdwani et al reported 33 patients from 15 Omani families, all with homozygous *DNASE1L3* variants, with a median onset of 4 years, ~60% lupus nephritis, and ~15% pulmonary hemorrhage, alongside recurrent urticarial rashes, hypocomplementemia, and a substantial burden of hypocomplementemic urticarial vasculitis features: arthritis (82%), urticarial vasculitis (79%), fever (49%), abdominal pain (36%), and conjunctivitis (25%).^[8]^

More recently, Aljaberi et al described 7 Emirati children from unrelated families carrying a novel p.Asn191Ser variant, functionally shown to impair *DNASE1L3* secretion and activity.^[9]^

Autosomal-recessive *DNASE1L3* deficiency was first described in 2011 in familial pediatric SLE, and since then, reports have documented a broad clinical spectrum ranging from SLE with severe lupus nephritis to hypocomplementemic urticarial vasculitis syndrome (HUVS). Recent cohort studies have expanded the number of genetically confirmed cases.^[4,6,8-11]^ Importantly, our study has several limitations that should be acknowledged. Molecular confirmation was unavailable for cases 2 and 4, and the DNASEI L3 variant identified in case 1 remains formally classified as a VIJS according to ACMG criteria. Therefore, case 1 cannot be considered definitively genetically confirmed DNASEI L3 deficiency. However, several findings support the clinical relevance of this variant, including homozygosity, parental consanguinity, early-onset disease, hypocomplementemia, nephritis, systemic inflammatory manifestations, and marked phenotypic overlap with previously reported DNASEI L3-associated disease. In addition, the presence of similarly affected siblings in both families strongly supports an underlying inherited monogenic autoimmune disorder. While cautious interpretation is warranted, genotype–phenotype concordance remains an important consideration in rare monogenic diseases, particularly in settings where functional studies or segregation analyses are not feasible.

Our series stands out in several important ways compared to previously published *DNASE1L3*-deficient cohorts. First, whereas most reports describe either a single affected child or one multiplex family,^[4,8-11]^ we uniquely report 2 unrelated sibling pairs from consanguineous backgrounds. This dual familial clustering provides a rare opportunity to directly compare intrafamilial similarities and differences, thereby enriching the understanding of genotype–phenotype variability in *DNASE1L3* deficiency.

Second, our cases illustrate a striking clinical heterogeneity that ranged from stable disease to fatal outcomes. Case 2 remained clinically stable on baricitinib for more than a year, whereas cases 1 and 4 succumbed to severe neurologic and renal complications, respectively. Such variability has been observed in prior reports, but rarely within such small sibling cohorts. Indeed, the 50% mortality in our series underscores the severe natural history of pediatric *DNASE1L3* deficiency, consistent with the refractory nephritis and fatal outcomes described in both the Omani cohort and isolated case reports.^[8,9,12]^

Another distinctive observation is the recurrence of ocular involvement, seen in three of our patients (cases 1–3), who developed conjunctival congestion, papilledema, or episcleritis-like inflammation. While systemic, renal, and cutaneous features dominate in the literature,^[3]^ ocular disease has been rarely emphasized, suggesting that our report highlights an underrecognized manifestation of *DNASE1L3* deficiency.

Equally important is the diversity of renal pathology. Renal involvement is a hallmark of *DNASE1L3* deficiency, most commonly presenting as class III or IV lupus nephritis^[4,8,9,13]^

Case 4 conformed to this pattern with biopsy-proven class IV lupus nephritis and an adverse outcome. In contrast, case 1 demonstrated IgA glomerulonephritis, an atypical finding that has not been widely reported in this context. This expands the recognized renal spectrum of *DNASE1L3* deficiency and indicates that nonclassical glomerulopathies may complicate diagnosis.

Our cases also revealed autoantibody heterogeneity. Case 2 was negative for ANA and anti-dsDNA, whereas case 4 displayed broad seropositivity, including ANA, dsDNA, anti-Smith, and ANCA antibodies. This variability mirrors published data in which *DNASE1L3* deficiency may present as either seropositive SLE or seronegative HUVS depending on age and disease stage.^[8,10,14,15]^ Such findings highlight the need to suspect *DNASE1L3* deficiency even when classical autoantibodies are absent.

Finally, case 2 demonstrated an apparent therapeutic response to baricitinib, a JAK1/2 inhibitor. Although *DNASE1L3*-specific therapeutic evidence remains limited,^[8]^ the interferon-driven pathogenesis of the disease makes JAK inhibition biologically plausible. Reports of JAK inhibitors in interferonopathies are emerging, but to our knowledge, our case represents one of the first documented instances of clinical benefit in a *DNASE1L3*-deficient child.^[6,16]^ This novel observation underscores the potential role of targeted therapies in managing this ultra-rare condition and warrants further investigation (Table 2).

**Table 2:** summarizes how our cases compare with the largest DNASE1L3-deficient cohorts and familial reports.

Feature	Our series (n = 4)	Omani cohort (n = 33) [3]	Emirati cohort (n = 7) [4]	Early reports (2011–2013) [1, 2,10]
Pedigree	Two sibling pairs; consanguinity	15 families; consanguinity universal	4 families; consanguinity universal	Multiplex families (Middle East, Europe)
Onset age	2.6–5 yr	Median 4 yr	Childhood (<5 yr)	Early childhood
Skin	urticarial/vasculitic rashes	Urticarial/vasculitic frequent	HUVS-like urticaria	Urticarial vasculitis
Ocular disease	3/4 cases (conjunctival congestion, papilledema)	Not emphasized	Not emphasized	Rarely reported
Complement	Low C3/C4 in 3/4	Hypocomplementemia common	Hypocomplementemia common	Hypocomplementemia hallmark
Autoantibodies	Mixed: ANA/dsDNA ±, ANCA+	Variable	Variable	Variable (SLE or HUVS-like)
Renal disease	IgA GN (Case 1), LN IV (Case 4)	~60% LN (mostly class III/IV)	Lupus nephritis frequent	LN common
Variant type	Homozygous VUS in case 1 (DNASE1L3) and homozygous pathogenic variant in case 3; others untested	Homozygous loss-of-function (founder variant)	Homozygous missense (p.Asn191Ser)	Multiple homozygous/compound heterozygous LoF variants
Therapy course	Steroids, ciclosporin, ACEI; case 2 stabilized on baricitinib; case 4 received cyclophosphamide + HCQ	Steroids, immunosuppressants (MMF, cyclophosphamide), biologics in refractory cases	Standard immunosuppressants; response variable	Steroids, cyclophosphamide, supportive; some biologics
Outcomes	Case 1: death (seizure); case 2: stable on JAKi; case 3: alive with chronic disease; case 4: death (renal failure)	Severe LN; some improved, others progressed; occasional mortality reported	Severe disease; some nephritis, long-term outcomes not fully detailed	Severe LN, vasculitis; some deaths reported

ACEI = angiotensin-converting enzyme inhibitor, ANA = antinuclear antibody, ANCA = anti-neutrophil cytoplasmic antibody, DNASE1L3 = deoxyribonuclease 1-like 3, GN = glomerulonephritis, HUV = hypocomplementemic urticarial vasculitis, JAKi = janus kinase inhibitor, IgA = immunoglobulin A, LN = lupus nephritis, MCV = mean corpuscular volum, MMF = mycophenolate mofetil, SLE = systemic lupus erythematosus, VUS - variant of uncertain significance.

## 4. Conclusion

Taken together, our cases underscore several important clinical implications. Genetic testing should be strongly considered in children presenting with an early-onset vasculitic rash, hypocomplementemia, and nephritis, even when ANA or anti-dsDNA antibodies are negative, as conventional serologies may not capture the full disease spectrum. Our findings also emphasize the importance of careful genotype–phenotype correlation in suspected monogenic lupus-spectrum disorders, particularly in patients with strong clinical features but incomplete molecular confirmation.

Moreover, the renal phenotype of DNASEI L3-associated disease appears broader than previously appreciated, encompassing not only the well-recognized forms of lupus nephritis but also atypical presentations such as IgA glomerulonephritis. Our observations also suggest that ocular involvement may be underdiagnosed and therefore deserves systematic screening in affected children. From a therapeutic perspective, the stabilization of one patient on baricitinib points to the potential role of JAK inhibitors in this interferon-driven disorder, although systematic evaluation is still required. Finally, the high mortality observed in our series highlights the urgent need for novel, targeted therapies addressing the DNASEI L3-extracellular DNA-interferon axis, which remains at the core of disease pathogenesis.A limitation of this study is that not all patients underwent comprehensive genetic confirmation, and one identified DNASEI L3 variant remains classified as a VUS according to ACMG criteria. Nevertheless, the combination of consanguinity, familial clustering, early-onset disease, hypocomplementemia, and strong phenotypic concordance supports the likelihood of an underlying inherited monogenic autoimmune disorder in these families. Further functional and genetic studies are warranted to better define the pathogenic role of such variants and to expand understanding of the clinical spectrum of DNASEI L3-associated disease.

## Author contributions

**Supervision:** Basheer Khalil.

**Writing – original draft:** Alyamama Kousa, Ahmad Alhamwi, Mohammad Khaled Alsayed, Sara Jabaly.

**Writing – review & editing:** Alyamama Kousa, Ahmad Alhamwi, Mohammad Khaled Alsayed, Sara Jabaly.
